# Antisense RNA decreases AP33 gene expression and cytoadherence by *T. vaginalis*

**DOI:** 10.1186/1471-2180-7-64

**Published:** 2007-07-03

**Authors:** V Mundodi, AS Kucknoor, JF Alderete

**Affiliations:** 1 Department of Microbiology, University of Texas Health Science Center, 7703 Floyd Curl Drive, San Antonio, TX 78229-3900, USA

## Abstract

**Background:**

Host parasitism by *Trichomonas vaginalis *is complex. Adherence to vaginal epithelial cells (VECs) is mediated by surface proteins. We showed before that antisense down-regulation of expression of adhesin AP65 decreased amounts of protein, which lowered levels of *T. vaginalis *adherence to VECs. We now perform antisense down-regulation of expression of the *ap33 *gene to evaluate and confirm a role for AP33 in adherence by *T. vaginalis*. We also used an established transfection system for heterologous expression of AP33 in *T. foetus *as an additional confirmatory approach.

**Results:**

We successfully select stable trichomonads with sense (S) and antisense (AS) plasmids. RT-PCR confirmed decreased amounts of *ap33 *mRNA in AS-transfected parasites, and decreased amounts of AP33 had no effect on growth and viability when compared to wild-type (wt) trichomonads. Immunoblots of proteins from AS-transfectants gave significant decreased amounts of functional AP33 capable of binding to host cells compared to wt- and S-transfected trichomonads. As expected, AS-transfectants had lower levels of adherence to VECs, which was related to reduction in surface expression of AP33. Stable expression of *T. vaginalis *AP33::HA fusion in *T. foetus *was confirmed by immunoblots and fluorescence. The episomally-expressed surface AP33::HA fusion increased adherence of trichomonads to human VECs, which was abrogated with anti-AP33 serum.

**Conclusion:**

These results using both antisense inhibition of gene expression and AP33 synthesis and the heterologous expression of AP33 in *T. foetus *confirms a role for this protein as an adhesin in *T. vaginalis*.

## Background

The protozoan *Trichomonas vaginalis *is responsible for the number one, non-viral sexually transmitted disease (STD) worldwide [1]. There are ~9 million new cases of vaginitis in the US alone [2-4]. Trichomonosis causes serious health consequences for women, including preterm delivery, low birth weight infants, infertility cervical cancer, pelvic inflammatory disease and infection by other STD agents [5-9]. Trichomonosis also predisposes humans to HIV by increasing the portal of entry and exit of virus [10]. A recent study showed a relationship between trichomonosis and prostate cancer [11]. Unlike other STDs, the prevalence of *T. vaginalis *does not decrease with age [12,13]. Given the significant human morbidity caused by *T. vaginalis*, there is an urgency towards identifying virulence factors, elucidating the mechanisms of pathogenesis, and developing interference strategies.

Adherence by *T. vaginalis *to vaginal epithelial cells (VECs) is preparatory for colonization and infection. Identification and characterization of five surface proteins (AP120, AP65, AP51, AP33 and AP23) involved in attachment to VECs has provided an understanding, in part, of the molecular basis of host cell adherence by this parasite [14-17]. The adhesins interact with host cells via ligand-receptor interactions [16-18]. Not surprisingly and as expected, there is a direct relationship between surface expression of adhesins and levels of cytoadherence [16,19]. All members of the adhesin gene families are coordinately up-regulated by iron [16,17,20], and iron appears important for compartmentalization and surface placement of the proteins [17]. Interestingly, the adhesins have sequence identity to metabolic enzymes located in the double membrane bound hydrogenosome organelle[18,21-24].

Adding to the complexity of these proteins is the fact that the genes encoding the adhesins are members of multigene families [14,21,22], which makes individual gene knockout approaches impractical for genetic-molecular studies. Therefore, a recent study by us used antisense technology as a genetic approach to confirm the importance of the prominent AP65 adhesin in adherence to VECs [17,25]. In addition, as an alternative approach to confirm AP65 function we established a transfection system for heterologous expression of the *T. vaginalis *AP65 in *T. foetus *and showed surface placement of AP65 that led to higher levels of *T. foetus *attachment to VECs [26]. In this report, we demonstrate a role of AP33 in parasite adherence to the host cells by antisense inhibition of *ap33 *expression. Furthermore, we show heterologous expression of AP33 on the surface of *T. foetus*, which elevated *T. foetus *cytoadherence.

## Results

### Plasmid construction and isolation of stable transfectants

**Figure 1:**
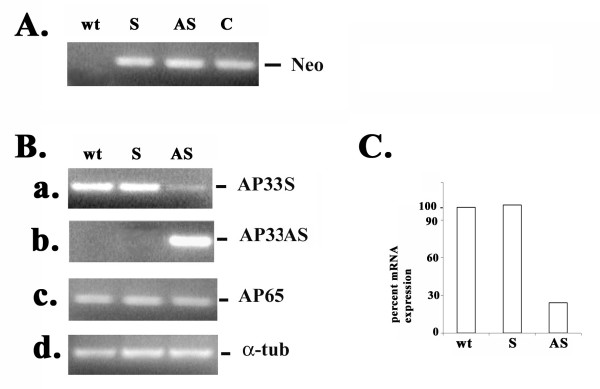
**Transfection and RT-PCR showing reduced *ap33 *mRNA levels in *T. vaginalis *trichomonads transfected with the antisense plasmid**. Part A shows PCR amplification of the *neo *coding region in transfected parasites. The ethidium bromide (EtBr)-stained band after electrophoresis in 1% agarose is the PCR product of the *neo *gene that was amplified using DNA from transfected *T. vaginalis *parasites. As expected, no PCR product was expected from wt organisms, and a predicted product was obtained from the plasmid used directly during PCR as a control (part A, lane labeled C). Part B gives bands after electrophoresis as in part A showing RT-PCR products for the *ap33 *S (panel a) and AS transcript (panel b). The RT-PCR product for *ap65*and *α-tubulin *are presented in panels c and d, respectively. Part C illustrates the quantitation of the transcript bands in part B (panel a) for the *ap33 *transcript in AS-transfected trichomonads compared to S-transfected and wt parasites. The bar graph shows the relative amounts of RT-PCR products for *ap33*. The amount of wild type *ap33 *transcript was normalized 100%. Quantification was done following Scion image β program.

We wanted to use the antisense approach to silence expression of *ap33*, as before [25]. Plasmid constructs containing the DNA fragment representing the coding region of the *ap33-1 *gene [24] in the sense (S) or antisense (AS) orientations were generated. Transfected *T. vaginalis *isolate T016 parasites were selected using 200 μg per ml G418, and resistant S- and AS-transfected trichomonads were cloned in soft agar. To confirm the presence of the plasmids in drug-resistant trichomonads, PCR was performed to amplify a 795-bp coding region of *neo *using template DNA from wild type (wt) organisms and S- and AS-transfectants. As expected, Figure 1A shows the *neo *PCR products in S- and AS-transfected trichomonads harboring the plasmids. No PCR product was obtained from wt organisms.

### Antisense mRNA modulates amounts of ap33

We next performed RT-PCR on total RNA isolated from S- and AS-transfectants and wt *T. vaginalis *as shown in Figure 1B. Results show the decreased intensity of the 580-bp PCR product of the *ap33 *coding region in the AS-trichomonads compared to S- and wt organisms (panel a). In contrast, the primers to the control *ap65 *gene gave equal intensities of 500-bp RT-PCR product (panel c), and this confirms specificity in the antisense inhibition of *ap33 *and the use of equivalent amounts of total RNA in the reactions. Only the AS-transfectants yielded a product from RT-PCR when specific primers were used to amplify a 525-bp product of the antisense transcript (panel b). As an additional control to show equal amounts of RNA, RT-PCR was performed to obtain a 650-bp product with primers specific to the *α-tubulin *gene (panel d), which is constitutively expressed in trichomonads. Finally, we performed the Scion image β program to quantitate RT-PCR products. Figure 1C shows an ~70% decrease in amounts of *ap33 *transcript (Figure 1B, panel a) when compared to the wt organisms and S-transfectants. As additional controls to show that transfection alone had no effect on *ap33 *mRNA levels, trichomonads were transfected with plasmid without insert and with plasmid carrying the *ap65 *antisense [25]. No effect on *ap33 *transcript levels by RT-PCR were seen for these controls. It is noteworthy that the primers used for RT-PCR of *ap33 *cross-hybridize with all three *ap33 *genes of the multigene family. Therefore, as all three *ap33 *genes are expressed under these growth conditions and given the decrease in mRNA by ≥ 70%, the RT-PCR data indicate that antisense RNA expression decreases the amounts of endogenous transcripts of all three *ap33 *genes.

### Antisense decreased amounts of AP33

**Figure 2:**
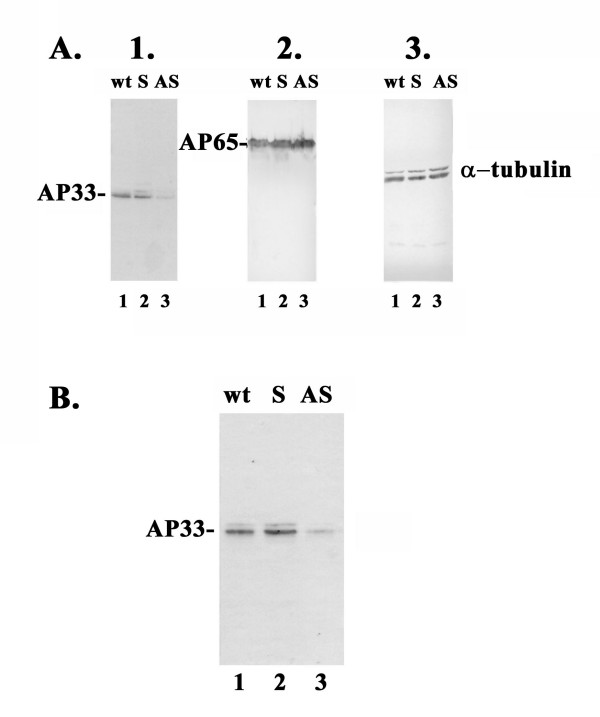
**Immunoblot analysis of S- and AS-transfected trichomonads and wt *T. vaginalis *organisms**. Part A presents triplicate blots of total protein from 10^7 ^trichomonads after SDS-PAGE on 10% acrylamide before blotting onto Hybond-P membranes. The blots were probed with mAb F5.2 to AP33 (panel 1), mAb 12G4 to AP65 (panel 2) [17], and mAb to α-tubulin (panel 3). Part B presents immunoblot results from a ligand assay, which was performed using extracts from equal numbers of organisms.

An immunoblot was performed using as probe monoclonal antibody (mAb) F5.2 to AP33 [24]. As shown in panel 1 of Figure 2A, the lanes with total proteins derived from equal numbers of wt (lane 1) and S-transfected parasites (lane 2) had readily detectable AP33 compared to AS-transfected parasites (lane 3). In contrast, the mAb 12G4 to AP65 shown in panel 2 had equal amount of protein for each sample in duplicate blots of panel 1, indicating that the effect of *ap33 *antisense RNA is specific. In panel 3 the mAb to α-tubulin reaffirms that equal amounts of protein was added to each lane and served as another internal control. Figure 2B presents an immunoblot using mAb F5.2 to AP33 after a ligand assay to measure amounts of functional AP33. There was decreased amounts of AP33 bound to VECs from extracts of AS-transfectants (lane 3) compared to wt (lane 1) and S-transfected parasites (lane 2). The amount of AP33 seen in lane 3 as determined by the Scion image β program was decreased by ~70%. This extent of inhibition of both mRNA seen above (Figure 1B, panel a) and protein synthesis is evidence that all three *ap33 *genes were down-regulated in expression.

### Reduction in amount of AP33 does not affect parasite growth

**Figure 3:**
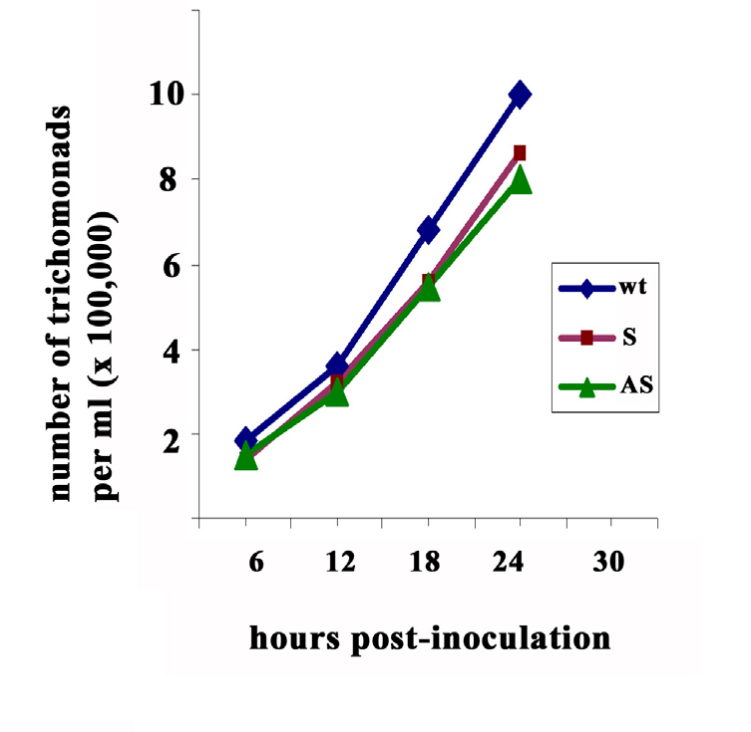
**Representative growth kinetics of wt organisms and S- and AS-transfected trichomonads cultivated in batch culture**. Medium was inoculated with 10^5 ^parasites, and cell densities at different time points were enumerated using a hemocytometer. Similar results were obtained from three growth experiments performed independently at different days.

Because AP33 is β-succinyl coenzyme synthetase also localized to the hydrogenosome where ATP is generated through the oxidation of pyruvate [27], we felt it important to determine whether inhibition of expression of this gene affected overall parasite growth and multiplication. As illustrated in Figure 3, there was no difference in the growth rates between wt and AS-transfected parasites nor was there any adverse effect on the viability and motility of trichomonads, as before [25].

### Decreased surface expression of AP33 and lower levels of adherence by AS-transfectants

**Figure 4:**
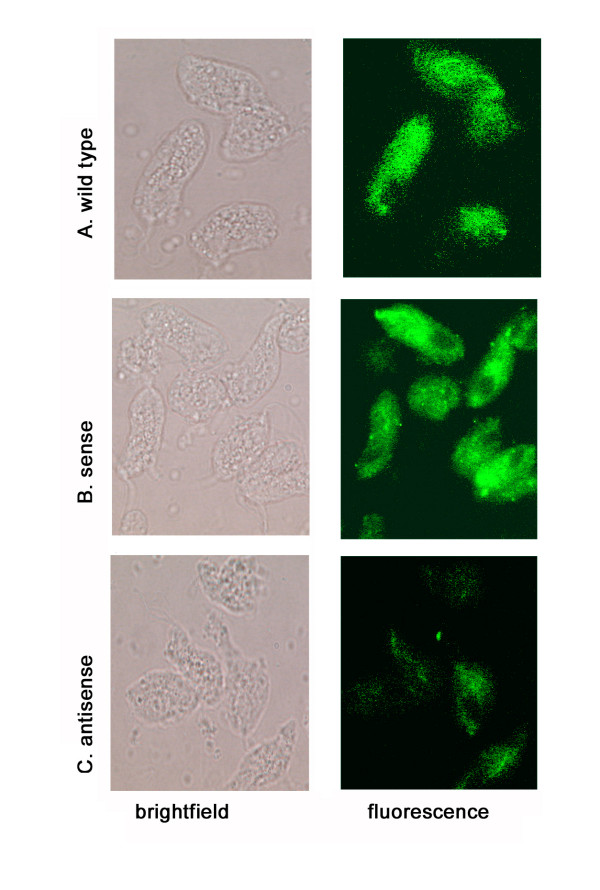
**Immunofluorescence showing decreased surface expression of AP33 in non-permeabilized, AS-transfected (panel C) compared to S-transfected (panel B) and wt trichomonads (panel A)**. Rabbit polyclonal anti-AP33 serum characterized previously [24] was used as described in Methods.

**Figure 5:**
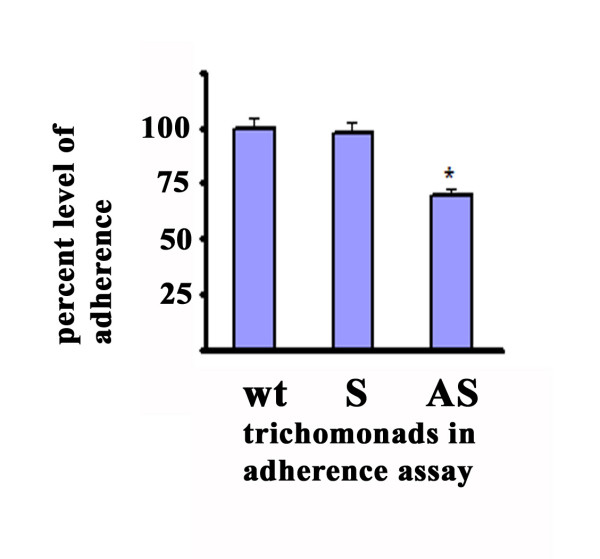
**Relative adherence of AS-transfectants (bar AS) compared with wt-(bar wt) and S-transfected (bar S) parasites**. *T. vaginalis *parasites expressing antisense display decreased adherence to MS-74 VECs. The extent of adherence by wt organisms was normalized to 100% for comparative purposes. The results are the average from four different experiments. Each experiment was performed in quadruplicate samples. The asterisk illustrates that this extent of decrease was statistically significant (p < 0.05).

Immunofluorescence experiments were then performed to examine surface expression of AP33. Figure 4 demonstrates the decreased intensity of fluorescence by polyclonal rabbit anti-AP33 serum antibody characterized previously [28] in the non-permeabilized AS-transfected organisms (panel C) compared with wt (panel A) and S-transfected organisms (panel B) handled identically. These result are in agreement with data presented above on the decreased amounts of total AP33 in AS-transfectants. Under no conditions was any fluorescence detected using normal rabbit serum antibody as a control (not shown). Furthermore, the level of adherence by AS trichomonads was measured and found to be ~30% lower than wt and S-transfected parasites (Figure 5), and this decrease in extent of adherence is not unexpected given the availability of alternative surface adhesins, such as the prominent AP65 adhesin [17], for cytoadherence. In separate control experiments performed simultaneously, trichomonads transfected with plasmid without insert and plasmid carrying the *ap65 *antisense had no effect on amounts of AP33. As with the S transfectants, no decrease in growth and adherence was evident by trichomonads transfected with plasmid without insert. Thus, these data suggest strongly that the effect was due to absence of surface AP33 and not to nonspecific events such as those that might result from transfection alone.

### Episomal expression of *T. vaginalis *AP33 elevates adherence levels in *T. foetus*

**Figure 6:**
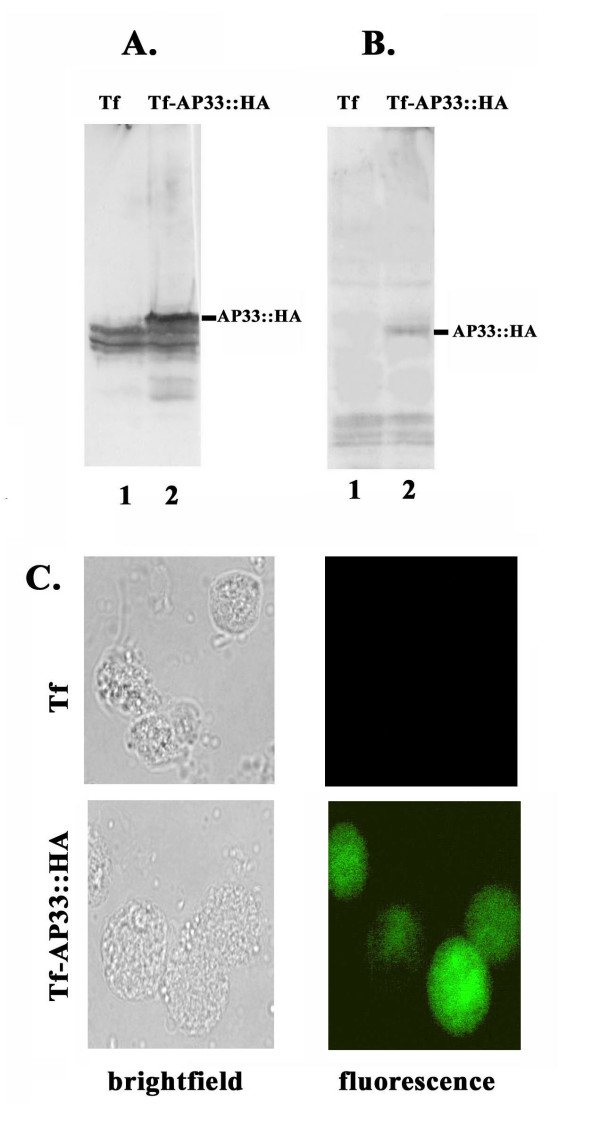
**Immunoblot and fluorescence analyses showing episomal expression of AP33::HA in *T. foetus***. A and B: Total protein from 10^7 ^parasites were separated on 10% SDS -PAGE and blotted on to Hybond-P membrane. The blots were probed with mAb F 5.2 to AP33 (A) and mAb to HA (B). C: Immunofluorescence was performed using anti-HA mAb on non-permeabilized wt (Tf) and transfected trichomonads (Tf-AP33::HA). Brightfield microscopy shows the same trichomonads in the field used for fluorescence microscopy.

As before [26], heterologous expression in *T. foetus *parasites is an additional approach for studying the function of *T. vaginalis *virulence genes. Therefore, *T. foetus *were transfected with the S plasmid to generate a fusion protein containing hemagglutinin (AP33::HA). Stable transfectants were obtained and characterized, also as before [26]. Episomal expression of AP33::HA was verified by RT-PCR (data not shown), and synthesis of AP33::HA was demonstrated by immunoblot analysis using mAb F5.2. Figure 6A (lane 2) shows the higher molecular weight AP33::HA fusion protein band in total protein blots of transfected *T. foetus*, but not wt *T. foetus *(lane 1). Bands common to lanes 1 and 2 illustrate the crossreactivity by mAb 5.2 with the *T. foetus *equivalent protein(s). Not unexpectedly based on our previous work [28], the lower-sized bands in lane 2 detected by mAb F5.2 are degraded AP33. Duplicate blots were also probed with mAb to HA (Figure 6B), and as expected, this mAb detected the fusion AP33::HA protein in transfectants (lane 2) but not in wt *T. foetus *(lane 1). The bands common to lanes 1 and 2 are nonspecific reactions by mAb with *T. foetus *proteins. The absence of detection of lower-sized, degraded AP33 in lane 2 is because the mAb is to HA, not AP33. We then performed fluorescence on non-permeabilized wt and AP33::HA transfected organisms. As can be seen in Figure 6C, the HA mAb only detected *T. foetus *trichomonads expressing the fusion protein, again providing evidence for the surface placement of AP33::HA. Finally and importantly and as before [26], *T. foetus *transfected with plasmid without insert gave results in all assay identical to those seen for wt *T. foetus*.

**Figure 7:**
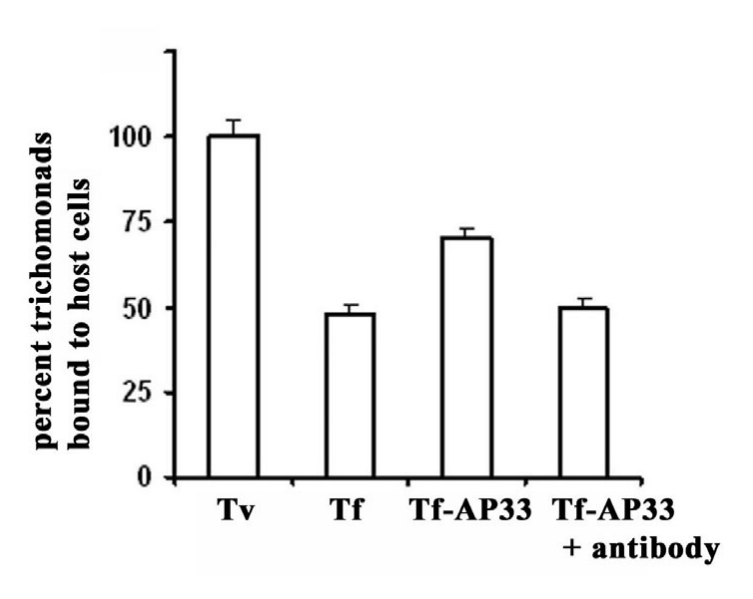
***T. foetus *with surface AP33::HA displays enhanced level of adherence to immortalized MS-74 VECs**. Transfected trichomonads with AP33::HA (bar labeled Tf-AP33) had higher levels of adherence compared to wt *T. foetus *(bar Tf), as before for heterologously-expressed AP65 [26]. The percent level of adherence was adjusted to that seen for *T. vaginalis *(Tv). Trichomonads expressing AP33::HA were pretreated with anti-AP33 antiserum (bar Tf-AP33 + antibody). The results are the average from four different experiments, and each experiment was carried out using quadruplicate samples.

Lastly, we performed an adherence assay for *T. foetus *expressing AP33::HA. As can been seen in Figure 7, episomal expression of AP33::HA (bar Tf-AP33) had elevated levels of adherence to VECs when compared to background levels seen for wt *T. foetus *(bar Tf). Levels of adherence was compared with those of *T. vaginalis *(bar Tv), which was normalized to 100%. Importantly, the increased level of adherence was attributed to surface AP33, as anti-AP33 antibody pretreatment of the transfectants (bar Tf-AP33 + antibody) gave levels of adherence equal to wt organisms (bar Tf). These experiments reinforce the idea that AP33 indeed is surface expressed and involved in the property of trichomonal adherence to VECs.

## Discussion

Our recent work with antisense RNA-mediated inhibition of expression of AP65 [25] coupled with the alternative approach of heterologous expression in the *T. foetus *trichomonad of AP65 [26] provided additional confirmatory evidence for a role of this surface protein in *T. vaginalis *adherence to VECs. These approaches are important to obtaining experimental evidence on a functional role for trichomonad virulence factors, such as adhesins. This is especially the case when virulence genes are members of multigene families [21-24], all of which are coordinately expressed [20-24] and in which individual gene knockouts may be difficult or impossible to achieve.

We now undertook similar characterization of AP33. AS-transfectants decreased in *ap33 *mRNA expression (Figure 1) had correspondingly less amounts of total and surface AP33 (Figures 2 and 4), which reflected lower levels of adherence to host cells (Figure 5). The fact that there was at best a 30% reduction in adherence to VECs in AS-transfectants is not problematic. These trichomonads have four additional surface proteins (AP120, AP65, AP51 and AP23) that provide redundant and alternative functions for adherence [14,17,23]. In fact, this extent of lowered levels of adherence is in agreement with previous data from inhibition experiments using anti-AP33 antibody and recombinant AP33 [24]. In addition, heterologous expression and surface placement of *T. vaginalis *AP33 (Figure 6) gave enhanced binding by *T. foetus *to human VECs (Figure 7), consistent with a recent report on heterologous expression of AP65 in *T. foetus *[26]. Altogether, this data affirms a role for AP33 in trichomonal adherence to host cells.

It is important to point out that the mechanism is unknown by which antisense inhibits gene expression in trichomonads. In our earlier work [25] and in this study we see decreased amounts of *ap33 *mRNA and AP33. As in other systems [29,30], translation arrest or degradation of mRNA following antisense-RNA-mRNA interactions may be occurring to account for the lower levels of *ap33 *transcript.

It was not surprising that decreased amounts of AP33 (α-SCS) did not affect overall trichomonal growth and multiplication (Figure 3) despite the fact that AP33 has sequence identity to a-succinyl CoA synthetase (α-SCS). No effect on parasite viability and growth parameters was also evident upon down-regulation of *ap65 *expression [25]. It is known that the parasites are capable of surviving without the hydrogenosome enzymes. For instance, MR100 is a drug-resistant isolate [31] lacking the adhesins-enzymes [17], and these parasites are unable to cytoadhere to VECs [17]. Indeed, parasites are capable of generating energy (ATP) through alternative metabolic pathways [32].

Recent reports [33,34] have suggested that trichomonad LPG may also be a mediator of adherence to host cells. LPG mutants showed a decreased ability to cytoadhere. These findings may suggest additional mechanisms by which the parasite successfully colonizes the host. Such redundancy is not surprising given the existence of multiple mechanisms by which pathogens target host cells and tissues [35]. In this report and an earlier paper [25] we decrease synthesis of two protein adhesins by antisense and, as predicted, show concomitant lower levels of adherence. These latter findings are also consistent with the numerous reports supporting a role for surface proteins as adhesins [15-18,20-26,28,36]. In addition, recent work on the relationship between polyamine metabolism and putrescine secretion on *T. vaginalis *adherence and cytotoxicity [36], while reinforcing the role of surface protein adhesins, illustrates the complexity of this adherence phenotype, which also involves host protein acquisition [37] and cysteine proteinases [38,39]. This work [36] establishes a testable hypothesis that has the potential to integrate the various adherence models. Therefore, further work will be needed to fully understand how these seemingly distinct mechanisms of adherence are integrated.

## Conclusion

These results using both antisense inhibition of gene expression and AP33 synthesis and the heterologous expression of AP33 in *T. foetus *confirms a role for this protein as an adhesin in *T. vaginalis*. Further, it is clear that antisense technology and heterologous expression in a different Trichomonas species are enabling experimental approaches to investigate and dissect the complex process of parasite adherence to host cells. This is especially crucial when other genetic approaches are untenable. Notwithstanding the possible existence of additional adherence mechanisms, the available data continue to support a role for surface AP33, and the other protein adhesins, in parasite recognition and binding to host cells.

## Methods

### Parasite culture and host cells

*Trichomonas vaginalis *isolate T016 and *Tritrichomonas foetus *(02–97) were grown in trypticase-yeast extract-maltose (TYM) medium with 10% heat-inactivated horse serum [40]. Immortalized MS-74 VECs used before [17,25] were grown in D-MEM (Invitrogen-Life Technologies, Carlsbad, CA) supplemented with 10 % fetal bovine serum at 37°C in presence of 5% CO_2_.

### Generation of sense (S) and antisense (AS) plasmids with ap33 coding region

The S and AS plasmids, designated pBS-*neo*-*ap33*-S and pBS-*neo*-*ap33-*AS, respectively, were constructed by cloning the coding region of *ap33 *gene in forward (S primer, 5'-CATACGCATATGCTCGCAGGCGACTTCTC-3' and AS primer, 5'-GATCTTGGTACCATTCTCTTCATCTCCTCG-3') and reverse orientation (S primer, 5'-CATACGGGTACCATGCTCGCAGGCGACTTCTC-3' and AS primer, 5'-GATCTTCATATGCCATTCTCTTCATCTCCTCG-3'). The original plasmid [41] was used to generate the plasmid used by us recently [25] to study the effect of antisense down-regulation of *ap65 *expression on adherence. This plasmid was modified as described [25] to replace the S and AS *ap65 *with the S and AS *ap33*. Briefly, after removal of the *ap65 *gene in the plasmid by partial digestion, the *ap33-1 *gene [24] of 0.9-kb was cloned into the *Nde*I and *Asp*718 sites. The authenticity of S- and AS-*ap33 *plasmids were confirmed by sequencing. Plasmid DNA for transfection was purified using maxi prep columns (Qiagen, Inc., Valencia, CA).

### Stable transfection and selection for G418 resistance

Transfection of *T. vaginalis *and *T. foetus *cells was carried out by electroporation [42]. Parasites at early logarithmic phase of growth were used for transfection. Briefly, 4 × 10^7 ^parasites were centrifuged at 1800 rpm at 4°C, and the pellet resuspended in 400 μl fresh TYM before transferring into a 4-mm gap cuvette (BTX^®^, Genetronics, Inc., San Diego, CA) with 25 μg of plasmid DNA. Electroporation was performed at 320 V, 1000 microfarads and 725 ohms using ECM 630 Electro cell manipulator (BTX^®^). Following the pulse, cells were placed on ice for 10 min and transferred into two T25 flasks with 50 ml of fresh TYM-serum medium. The cells were grown free of drug for 24 h followed by the addition of Geneticin (G418) (Invitrogen) at 200 μg ml^-1^. Single cells were cloned using soft-agar plates containing 25 μg ml^-1 ^G418. Four different clones were further analyzed, and representative data from one of the clones is presented in this report. The DNA was isolated from single cell cultures using DNAzol (Invitrogen) and further purified by phenol-chloroform extraction. The presence of plasmid in numerous single cell clones was confirmed by PCR amplification of the *neo *gene using the *neo*-sense primer 5'GATCGGTACCATGATTGATTGAACAAGATGGATTG-3' and *neo-*antisense primer5'CTTTAGACCAAGTTCGTGTCAGAAGAACTCGTCAA-G-3, as shown in Figure 1A.

### RNA isolation and RT-PCR analysis

Total RNA was isolated from both wt and transfected parasites using the Trizol reagent (Invitrogen). For RT-PCR, 1 μg of total RNA was reverse transcribed using SuperScript II RNase H^- ^Reverse Transcriptase (Invitrogen). Then, 10% of the reverse transcribed cDNA was used as template for the PCR reactions. The primers used for PCR amplifications of the *ap33 *transcript were as follows: *ap33*-sense primer, 5'CTCATTTTCGTCCCAGCTCC-3' and *ap33*-antisense primer, 5'AAACAATACCGATCTTACCG-3'. For *α-tubulin *the sense primer was 5'-ACTCTGCTGCCTCGAGCACGGTATC-3', and antisense primer was 5'-GAAATGACTGGTGCATAAAGAGC-3'. To demonstrate the synthesis of AP33 antisense transcript, total RNA was reverse transcribed using a gene-specific primer (5'CATACGCATATGCTCGCAGGCGACTTCTC-3'). The forward and reverse primers for PCR of the antisense transcript were 5'CTCATTTTCGTCCCAGCTCC-3' and 5'AAACAATACCGATCTTACCG-3', respectively.

### Immunoblot detection of AP65, AP33 and AP33::HA

Total proteins of 10^7 ^*T. vaginalis *and *T. foetus *organisms were obtained as before using trichloroacetic acid (TCA) [43] for sodium dodecylsulfate-polyacrylamide gel electrophoresis (SDS-PAGE) [44] prior to blotting onto Hybond-P membranes (Amersham) for immunoblot detection with mAb 12G4 to AP65, mAb F5.2 to AP33, and mAb to hemagglutinin (HA) (Sigma) for detection of the fusion AP33-HA. TCA-precipitated proteins were solubilized using electrophoresis dissolving buffer [44]. SDS-PAGE was carried out using 10% acrylamide gels. The mAbs and epitope reactivity have been described before [17,24]. Following reactivity with the mAb probes, the bands were visualized by the chemiluminescence assay with horseradish peroxidase as the color developer (BioRad Laboratories, Hercules, CA).

### Ligand assay to assay for functional AP33

The ligand assay to detect adhesins that bind the host cells was carried out as before [16]. Briefly, after fixation and processing of HeLa cells with glutaraldehyde, 10^6 ^*T. vaginalis *cells were incubated with a trichomonal detergent extract derived from 2 × 10^7 ^solubilized parasites. After incubation, cells were vigorously washed to remove unbound and loosely-associated trichomonad proteins. Cells were boiled in electrophoresis dissolving buffer to elute the HeLa cell-binding proteins followed by SDS-PAGE in 10% acrylamide. The gels were further stained with Coomassie brilliant blue for visualization, and duplicate gel was blotted onto Hybond-P membrane for immunoblot analysis using the mAb F5.2 to AP33.

### Surface AP33 detected by fluorescence

Immunofluorescence of AP33 on the surface was carried out using a modification of a recently-described procedure [17]. Briefly, 1 × 10^6 ^logarithmic phase organisms were washed twice with cold PBS and fixed with 4% paraformaldehyde for 30 min at RT. Fixed non-permeabilized organisms were washed in PBS, then blocked with 5% BSA for 1 h at RT prior to incubation for 1 h at RT of *T. vaginalis *with rabbit polyclonal anti-AP33 serum diluted 1:100 (v/v). Parasites were washed with PBS and incubated for 1 h at 37°C with fluoresceine isothiocyanate-conjugated anti-rabbit IgG (Sigma) diluted 1:100. Finally, parasites were washed twice with PBS and observed under 100× magnification using the Olympus BX41 microscope. For both wt *T. foetus *and *T. foetus *transfected with the plasmid encoding the AP33::HA fusion protein, fluorescence was performed similarly except that the mAb to hemagglutinin (HA) diluted 1:100 (v/v) was used.

### VEC adherence assay

Immortalized MS-74 human VECs [17,45] were used for adherence experiments. Confluent monolayers of MS-74 VECs on individual 96-well microtiter plates were stabilized with 3% glutaraldehyde, as before [16], prior to addition of labeled parasites. Late logarithmic growth phase parasites (5 × 10^6^) of *T. vaginalis *and *T. foetus *were washed in PBS and suspended in 1 ml of TYM without serum for labeling with calcein for 30 min at 37°C. Labeling was followed washing with PBS, and washed parasites (2.5 × 10^5^) were added to individual wells of confluent, fixed VECs. After plates were incubated at 37°C for 30 min, the wells were washed three times with PBS. The adherent parasites were lysed with 200 μl of 0.1% Triton X-100. The level of adherence to VECs was determined by the intensity of fluorescence of the lysate at excitation absorbance of 485 nm and emission wavelength of 528 nm with Synergy™ HT Multi-Detection Microplate Reader (Bio-Tek Instruments, Inc., Winooski, VT). For adherence assays using wt and *T. foetus *transfected with the plasmid encoding the AP33::HA fusion protein, trichomonads were also pretreated with anti-AP33 serum (1:100; v/v) prior to the adherence assay. This was done to insure that the increase in adherence by *T. foetus *was due to surface AP33::HA, as before [26].

### Reproducibility of experiments

Unless otherwise stated in the text, all experiments were performed numerous times and no less than on three different occasions.

## Abbreviations

AP65, adhesin protein of molecular weight 65-kDa; AP33, adhesin protein of molecular weight 33-kDa; AS, antisense; AP33::HA, fusion protein of AP33 and hemagglutinin (HA), BSA, bovine serum albumin; mAb, monoclonal antibody; PBS, phosphate buffered saline; SDS-PAGE, sodium dodecylsulfate polyacrylamide gel electrophoresis; RT, room temperature; S, sense; Tf, *Tritrichomonas foetus*; Tv, *Trichomonas vaginalis *VEC, vaginal epithelial cell

## Authors' contributions

VM carried out the design of the study and performed plasmid constructions, RT-PCR, immunoblots, ligand assay, growth curve, adherence assay and drafted the manuscript. ASK performed transfection and immunofluorescence. JFA participated in the design of the experiments, offered suggestions during the experiments, and helped to write the manuscript. All the authors read and approved the final manuscript.
